# Diversity and Composition of the Leaf Mycobiome of Beech (*Fagus sylvatica*) Are Affected by Local Habitat Conditions and Leaf Biochemistry

**DOI:** 10.1371/journal.pone.0152878

**Published:** 2016-04-14

**Authors:** Martin Unterseher, Abu Bakar Siddique, Andreas Brachmann, Derek Peršoh

**Affiliations:** 1 Ernst-Moritz-Arndt Universität Greifswald, Institut für Botanik und Landschaftsökologie, Greifswald, Germany; 2 Biozentrum der LMU München, Bereich Genetik, Martinsried, Germany; 3 Ruhr-Universität Bochum, AG Geobotanik, Bochum, Germany; Leibniz Institute DSMZ-German Collection of Microorganisms and Cell Cultures, GERMANY

## Abstract

Comparative investigations of plant-associated fungal communities (mycobiomes) in distinct habitats and under distinct climate regimes have been rarely conducted in the past. Nowadays, high-throughput sequencing allows routine examination of mycobiome responses to environmental changes and results at an unprecedented level of detail. In the present study, we analysed Illumina-generated fungal ITS1 sequences from European beech (*Fagus sylvatica*) originating from natural habitats at two different altitudes in the German Alps and from a managed tree nursery in northern Germany. In general, leaf-inhabiting mycobiome diversity and composition correlated significantly with the origin of the trees. Under natural condition the mycobiome was more diverse at lower than at higher elevation, whereas fungal diversity was lowest in the artificial habitat of the tree nursery. We further identified significant correlation of leaf chlorophylls and flavonoids with both habitat parameters and mycobiome biodiversity. The present results clearly point towards a pronounced importance of local stand conditions for the structure of beech leaf mycobiomes and for a close interrelation of phyllosphere fungi and leaf physiology.

## Introduction

Fungal leaf endophytes are a major component of the plant microbiome [1]. Since these fungi are highly diverse in species, ecology and functions a precise classification according to their preferred host plants, their location and interaction or their mode of transmission is advocated (reviewed by [2–4]). Systemic endophytes in grasses for example are known to strongly influence host resilience against herbivory and microbial pathogens [5]. Endophytes in perennial, woody plants are more diverse than in grasses and a distinction between the phyllosphere, rhizosphere, and lignosphere is generally recommended [3].

Diversity and functions of endophytes from different tissues are overlapping [6, 7] and generally influenced by various abiotic and biotic factors at regional [8] and local scales [9, 10]. The structure of surrounding vegetation [11] influences endophytic communities as well as host species identity [12–15], host genotype [16, 17] and other biotic and abiotic factors [18–22]. Finally, endophytes are strongly influenced be co-occurring microbes which often leads to complicated community dynamics [1].

Fungal leaf endophytes have traditionally been studied with cultivation-based approaches [23–26] including surface sterilisation to kill all superficially occurring microorganisms [27]. Cultivation approaches are time consuming, expensive and suffer from several biases, in particular from undersampling [4]. Culture-based methods often exclude biotrophic and slow growing species and favour the detection of rapidly growing species, although this bias could be reduced partially by improved cultivation methods [28, 29].

In recent years cultivation-independent next generation sequencing (NGS) approaches (i.e. metabarcoding of fungi) have opened new perspectives in fungal biodiversity research [30–35]. They are generating ever increasing amounts of sequence data [36, 37] and the accuracy of bioinformatics pipelines (e.g. [38]) and reference data bases [39, 40] are constantly improving.

The present study is part of a larger research project, which aims at broadening the understanding of leaf mycobiome dynamics under differing climatic conditions at reduced environmental complexity (http://gepris.dfg.de/gepris/projekt/245215303?language=en, last accessed 03/2016). To achieve this we established an experimental field sites in a natural, beech dominated mountain forest in southern Germany by planting young beech trees from a commercial tree nursery in northern Germany (termed "phytometer" trees) at two differing altitudes. For the present contribution, we analysed the as-is state of the phytometer leaf mycobiome immediately after planting at the two differently elevated experimental plots, thus reflecting its original constitution at the intensively managed nursery garden. We then compared its fungal biodiversity with that from naturally growing neighbouring beech trees.

We posited that phyllosphere mycobiomes respond to the local stand conditions of their host trees and therefore differ between the phytometer and natural trees (hypothesis 1). It was shown recently that mycobiome composition of the natural trees also differed on a smaller spatial scale between the valley and two mountain samples [41]. These results were partly recapitulated and related to the phytometer samples, which were expected to lack these patterns. We further aimed at answering the questions how biochemical properties of tree leaves differ between differing stand conditions of the trees, and if the measured biochemical constitution of leaves correlates with mycobiome diversity and composition.

## Materials and Methods

### Investigation site, phytometer experiment and field work

Establishment of the experimental sites and field work was achieved with full agreement of the Forstamt Berchtesgaden of the Bayerische Staatsforsten. Further permissions were not necessary because all activities took place outside protected or private areas and did not involve any endangered or protected species

**Fig 1:**
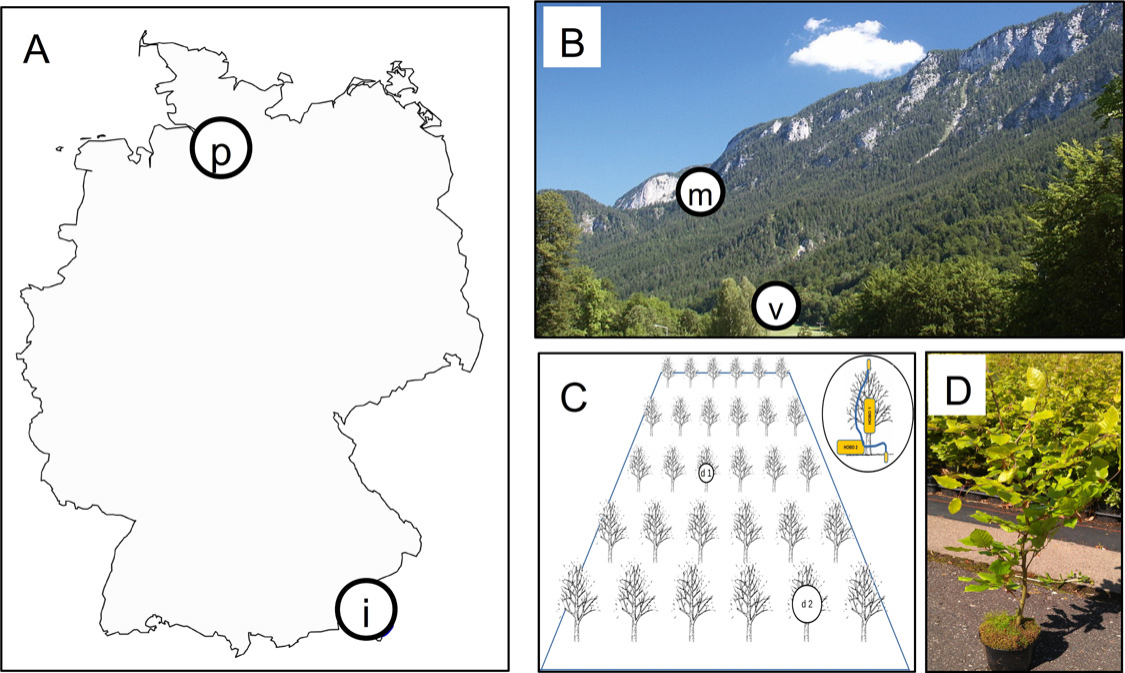
Origin of leaf samples and experimental design of the "phytometer" plot. [A] shows a map of Germany with the origin of the phytometer trees ("p"-labelled circle, Baumschule Hans Reinke GmbH in Rellingen near Hamburg, Germany) and the location of the major investigation area ("i"-labelled circle, Marktschellenberg, Bavaria, Germany). [B] Leaves of naturally grown trees were sampled in two locations at different altitudes of the investigation area ("v"-labelled valley site and "m"-labelled mountain site). This image of the Untersberg has been released by its author TomK32 into the public domain of German Wikipedia (https://commons.wikimedia.org/wiki/File:Untersberg-Westseite_von_Bischofswiesen_aus.jpg, last accessed 04/2016). The phytometer sites were established there, too. [C and D] The phytometer trees were planted in each altitude and two data logger modules (d1 and d2) were installed.

Two investigation sites with comparable edaphic parameters but different altitudes were selected in the mountain massif “Untersberg” in the Berchtesgaden Alps near the city of Marktschellenberg, Bavaria, Germany (Fig 1). One site was established at 975 m a.s.l. (‘mountain site’; Lat: 47.683158 Long: 13.002102), the second site is located at 515 m altitude and represents more moderate growth conditions for the tree species (‘valley site’; Lat: 47.712946 Long 13.040101). Both sites were selected in areas free of old-growth canopy trees. Pararendzina = Rendzic Leptosols (LPk) over gravel dolomite is found as soil type at both sites. It is humus-rich with a well developed topsoil layer (A horizon) at the valley site, while the A horizon is only weakly developed at the mountain site. The ground and understory vegetation of the mountain site was mainly composed of *Acer pseudoplatanus*, *Picea abies*, *Daphne mezereum*, *Cardamine* (= *Dentaria*) *enneaphyllos*, *Helleborus niger* and *Hepatica nobilis*. At the valley site, ground vegetation was different with dominance of *Acer pseudoplatanus*, *Mentha* spp., *Petasites hybridus*, *Equisetum sylvaticum* and *Rubus* sp. Between October 2013 and October 2014, local climate data loggers (see below) recorded an average temperature of 9.0°C (-7.6 to 36.4°C) and an average relative humidity of 93.9% (28.0 to 100) at the valley site and 7.6°C (-5.6 to 38.8°C) and 91.4% (20.8 to 100) at the mountain site. The total number of days with closed snow cover was 78 for the valley site and almost twice as much (146 days) for the mountain site. This period was free of weather extremes in that area, the observed differences between valley and mountain site can be therefore regarded as representative measurements.

At both sites, an area of approx. 20 m × 20 m was cleared from understorey woody and herbaceous plants. Within each of these two plots, 100 two-years old beech trees ("phytometer trees") were planted with bare roots into the natural soil in the beginning of October 2013 and fenced against damage by herbivorous animals (e.g. deer). At the time of planting, the trees were in vigorous conditions possessing healthy, fully green leaves with minimal damage. The phytometer trees were originally grown in a tree nursery in northern Germany (“Hans Reinke Baumschulen” in Rellingen near Hamburg, Fig 1A) where they experienced optimal growth conditions. The phytometer trees germinated from seeds originating from the Lusatian lowland area (Central Eastern Germany, HkG 81005). Thus the phytometer trees were expected to have no genetic pre-adaptations to the local conditions to which they were transferred. Each phytometer site was equipped with two data logger modules measuring relative humidity and temperature (HOBO Pro v2 U23; Hobo 1 in Fig 1C) and with two data loggers recording temperature with two external sensors (HOBO Pro v2 U21; Hobo 2 in Fig 1C). One sensor of Hobo 2 was fixed at the soil surface, the second in the canopy of the young trees at about 40 cm above the ground.

One day after planting, healthy looking green leaves without signs of ageing or damage were randomly sampled from five randomly chosen phytometer trees at each site and in the same manner from neighbouring naturally growing trees. Natural trees were older than the phytometer trees (ca. 15 cm diameter at breast height), their sampled leaves were in similar conditions as assessed with the naked eye and removed from locations as close to the forest floor as possible. Physiological key parameters of the sampled vital leaves were assessed by measuring area-based chlorophyll (surrogate for photosynthesis rate and leaf proteins) and flavonoid contents (surrogate for leaf phenols) with a non-invasive hand-held optical sensor clip (Dualex Scientific, Force-A, France). Arithmetic means of four measures per leaf (base, tip, right, left; all upper side) and ten leaves per tree were used to correlate leaf biochemistry with fungal diversity patterns (see below). Those ten leaves were combined and subsequently treated as one environmental sample. Leaf material was submerged into 70% ethanol for three minutes immediately after these measurements to kill or at least inhibit activity of epiphyllous microbiota during transport. The samples were stored in paper bags at 4–7°C. Within 48 h after sampling, leaves were thoroughly surface sterilised according to standard methods [42] and frozen at -80°C until further processing.

### DNA extraction and preparation of the Illumina amplicon library

Samples were thawed and homogenized in sterile distilled water with a commercial blender, then filtered through analytical sieves in order to separate differently sized particles. This procedure was similar to the first part of dilution-to-extinction cultivation [29]. Approximately 100 μg (fresh weight) of the 100–200 μm ø sized particles were used for DNA extraction with the Charge Switch® gDNA Plant Kit (Invitrogen) following the manufacturer's protocol. The remaining particle mass was permanently stored at -80°C.

A nested PCR approach was used for preparation of the Illumina amplicon library. First, the entire internal transcribed spacer (ITS) region was amplified with the universal primer pair V9G / ITS4 under standard conditions [43, 44], followed by clean-up of the amplicon mix with ExoSap-IT (Affymetrix UK Ltd., United Kingdom) according to manufacturer's instructions except that a 1:5 dilution of the reagents was used. The purified ITS amplicons then served as template DNA for the first Illumina-specific amplification of the full-length ITS rRNA gene region (see online supporting material "S1 Notes").

We used custom-made primer pairs (Microsynth AG, Switzerland) consisting of the commonly used ITS1F and ITS4 sequences, barcoding tags of varying length and 21bp of the Illumina sequencing primer. The latter served as binding site for the second library PCR primer pairs, which contained further barcodes for multiplexing purposes and the Illumina-specific anchor sequences[41]. PCR reactions also followed [41]. Final amplicons were visualised in 1% agarose gels stained with ethidium bromide

Band intensity was calculated from the gel images with ImageJ [45]. Amplicons were pooled in equimolar concentrations such that samples with lower band intensity were added in proportionally larger volumes than samples with stronger band intensity. The final amplicon mix was purified with Dynabeads Sequencing Clean Up Kit (Life Technologies GmbH, Germany) and shipped to the Genetics Sections, Biocenter of the LMU Munich, Germany. There the library was finalised for sequencing according to the manufacturer’s protocols (http://supportres.illumina.com/documents/documentation/system_documentation/miseq/preparing-libraries-for-sequencing-on-miseq-15039740-d.pdf). Paired-end sequencing of 2 x 300 nt with two additional 8 nt index reads and 3μl of the library was performed on an Illumina MiSeq platform (Illumina Inc.).

### Sequence processing bioinformatics

Raw forward (R1) and reverse (R2) reads were demultiplexed by the Illumina sequencer software according to their index combinations (501–701, 501–702, etc.) and adapters, indices and sequencing primers were removed. All reads thus started with forward (R1) and reverse tags (R2), respectively.

The study-specific bioinformatics workflow consisted of a second demultiplexing step to separate all tag combinations followed by quality filtering [46], chimera checking [40], ITS trimming [47], grouping of operational taxonomic units (OTUs) at 97% sequence similarity and taxon assignment [39]. as detailed in [41]. The bioinformatics are detailed in [41] and available as commented online supporting material ("S1 Text"). As an intermediate outcome, sequences were separated into ITS1 (stemming from the forward R1 reads) and ITS2 (from the reverse R2 reads). Generally lower phred scores were observed for R2 reads. Therefore the curated and demultiplexed data contained much less ITS2 than ITS1 reads (data not shown). Since a detailed comparative biodiversity analysis of both DNA markers was beyond the scope of this study, subsequent analyses were performed with the larger ITS1 data set.

The raw Illumina reads are provided under the NCBI SRA Accession SRX1211311.

### Biodiversity analysis

A spreadsheet master data file was compiled with two major output files (the final OTU table and the representative sequence FASTA file; see "S1 Table"). These data were conservatively curated by removing unique OTUs (OTUs occurring in only one sample regardless of the number of reads) and OTUs with less than 5 reads over all samples (for justification see [48]). Additionally, non-fungal OTUs (OTUs returning “no blast match” after automatic taxon assignment against the UNITE data set [39]), were discarded.

Fungal diversity was analysed with Fisher's alpha (a richness index, representing all species in a data set), Shannon index (considering both richness and abundance) and the two Hill numbers from Hill's series of diversity (N1 = exponent of Shannon index, representing "common" species and N2 = inverse Simpson index, representing "abundant" species according to [49]). Diversity was visualised using Box-Whisker plots. Analyses were combined with statistical tests (ANOVA of the multivariate generalized linear models) and randomised species accumulation curves [50, 51].

In order to account for unequal library size (differing sequencing depth per sample), which might bias diversity calculations, we additionally applied downsampling (rarefying) to the lowest read number per sample (here 9500) prior to diversity assessments.

Nonmetric multidimensional scaling (NMDS) and principal coordinate analysis (PCO) based on Bray-Curtis dissimilarities of square root-transformed read abundances were used to visualise community composition. The distinctiveness of leaf mycobiomes in different sub-datasets (i.e. phytometer vs. natural or valley vs. mountain) was tested with a permutational multivariate analysis of variance using distance matrices (PERMANOVA). In addition we used multivariate ("multispecies") generalized linear models (GLM) to test for significant correlations of the community data with environmental parameters [34, 52, 53].

The entire biodiversity analysis was performed in R (available freely on https://www.r-project.org/, last accessed 03/2016), the corresponding commands and all necessary data files (e.g. metadata) are available as online supporting material ("S2 Notes").

## Results

### Basic sequence data and diversity

**Fig 2:**
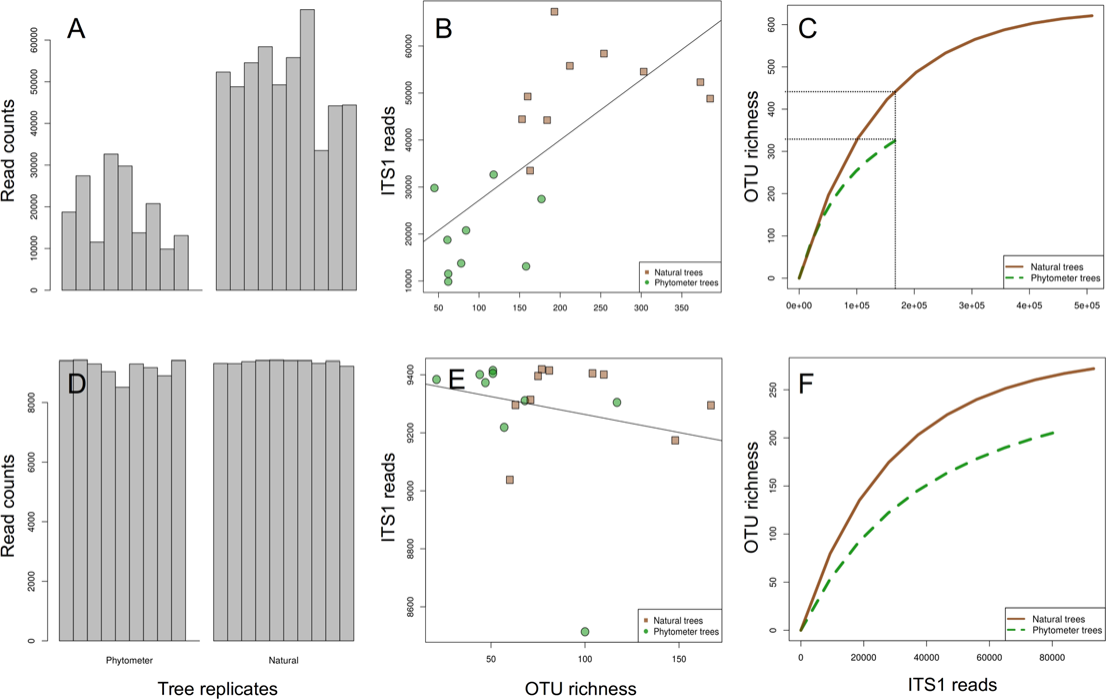
Read abundance and fungal OTU richness. [A-C] for the full data set and [D-F] for a smaller data set after rarefying (randomised downsampling) to 9500 reads per sample. [A and D] display read abundances of all natural and phytometer samples after data curation (removal of rare and unassigned OTUs). [B and E] display the interrelations between read abundance and OTU richness. Phytometer samples had lower richness values than natural samples irrespective of sequencing depth. [C and F] display randomised species accumulation curves allowing the comparison of OTU richness at an identical sequencing effort. For both data sets the leaf mycobiome of phytometer trees revealed poorer richness than of natural trees.

The demultiplexing, quality filtering and OTU clustering resulted in 686165 ITS1 reads separated into 697 OTUs from 19 samples (one sample from the phytometer trees failed during library preparation). Significantly fewer reads were obtained from phytometer samples than from the natural trees (Fig 2A, mean read number = 17762 vs. 50855, t-test t = -7.65, p < 0.05).

Read abundance and OTU richness were strongly positively correlated (Fig 2B, adjusted R^2^ = 0.48, p < 0.05). After decoupling OTU richness from read numbers with randomised species accumulation curves, natural samples revealed clearly higher fungal OTU richness than phytometer trees (Fig 2C, 334 observed OTUs for phytometer trees, 448 OTUs for natural trees at an identical sampling intensity of ca. 180000 reads). After randomised downsampling to an identical sequencing depth per sample (Fig 2D), read abundance and OTU richness were no longer correlated (adjusted R^2^ < 0.01, p = 0.38; Fig 2E), whereas the higher fungal OTU richness for natural trees remained statistically significant (t-test: t = - 2.23, p = 0.04, mean phytometer richness = 62 OTUs per sample; mean natural richness = 96 OTUs per sample; Fig 2E and 2F)

**Fig 3:**
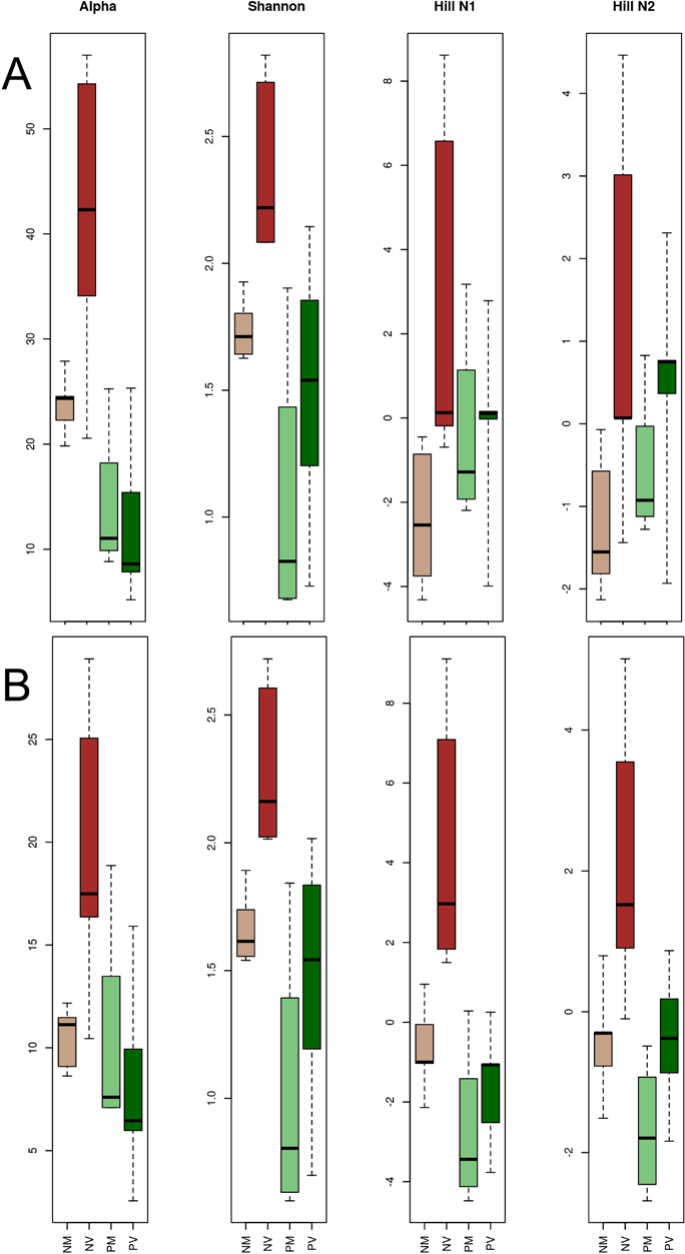
**Diversity indices for the full [A] and rarefied [B] data set.** A general trend of higher fungal diversity is displayed for natural samples (NV = natural valley, NM = natural mountain) at least for alpha and Shannon diversity [compare Table 1]. The leaf mycobiome of natural valley samples (NV) displayed the highest diversity values. All four indicators were significantly higher than those of natural mountain samples (NM). For both data sets, the factor elevation obtained insufficient statistical support for phytometer samples (PV = phytometer valley, PM = phytometer mountain) but resulted in statistical significance within natural trees.

All diversity measures resulted in higher diversity values for natural than for phytometer samples (Fig 3, Table 1) with higher statistical confidence for the rarefied data (Fig 3B, Table 1). Leaves from natural valley trees consistently displayed the highest fungal diversity. Fungal diversity varied for the other three groups, depending on the used data set and the applied diversity measure (Fig 3). The corresponding statistical tests calculated significant differences between phytometer and natural leaf mycobiomes (factor "Setting"). When testing against the parameter "elevation", test statistics were insignificant for phytometer samples, but yielded significant differences for the natural trees (Table 1).

**Table 1:** Basic diversity statistics based on a multispecies generalized linear model. Results show significant differences between natural and phytometer leaf mycobiomes (factor "Setting") for the full as well as for the downsampled (rarefied) data. The significant diversity values (printed in bold for p < 0.05) for the parameter elevation were entirely driven by the natural samples, since the phytometer leaf mycobiomes did not reveal any significant differences between valley and mountain site.

		Fisher			Shannon			Hill N1			Hill N2		
		Sum of squares	dev	p	sum of squares	dev	p	sum of squares	dev	p	sum of squares	dev	p
Setting	full data	1809	13.5	**0.002**	2.76	3.10	**0.014**	89	8.89	**0.004**	15	4.17	**0.043**
	rarefied data	169	5.33	**0.031**	2.5	3.1	**0.017**	71	7.42	**0.014**	14	3.97	0.056
Elevation	full data	359	2.05	0.225	1.41	3.44	**0.014**	68	10.49	**0.004**	23	6.15	**0.007**
	phytometer	5.46	0.11	0.79	0.42	2.09	0.108	6	2.03	0.178	6	1.47	0.171
	natural	799	7.34	**0.025**	1.03	1.7	**0.005**	81	8.61	**0.009**	18	4.72	**0.021**
	rarefied data	70	1.23	0.313	1.41	3.44	**0.011**	57	9.66	**0.005**	21	5.62	**0.01**
	phytometer	10	0.72	0.462	0.44	2.09	0.093	6	1.93	0.184	6	1.47	0.142
	natural	210	7.43	**0.019**	1.01	1.7	**0.001**	66	7.88	**0.009**	17	4.19	**0.034**

### Community composition

**Fig 4:**
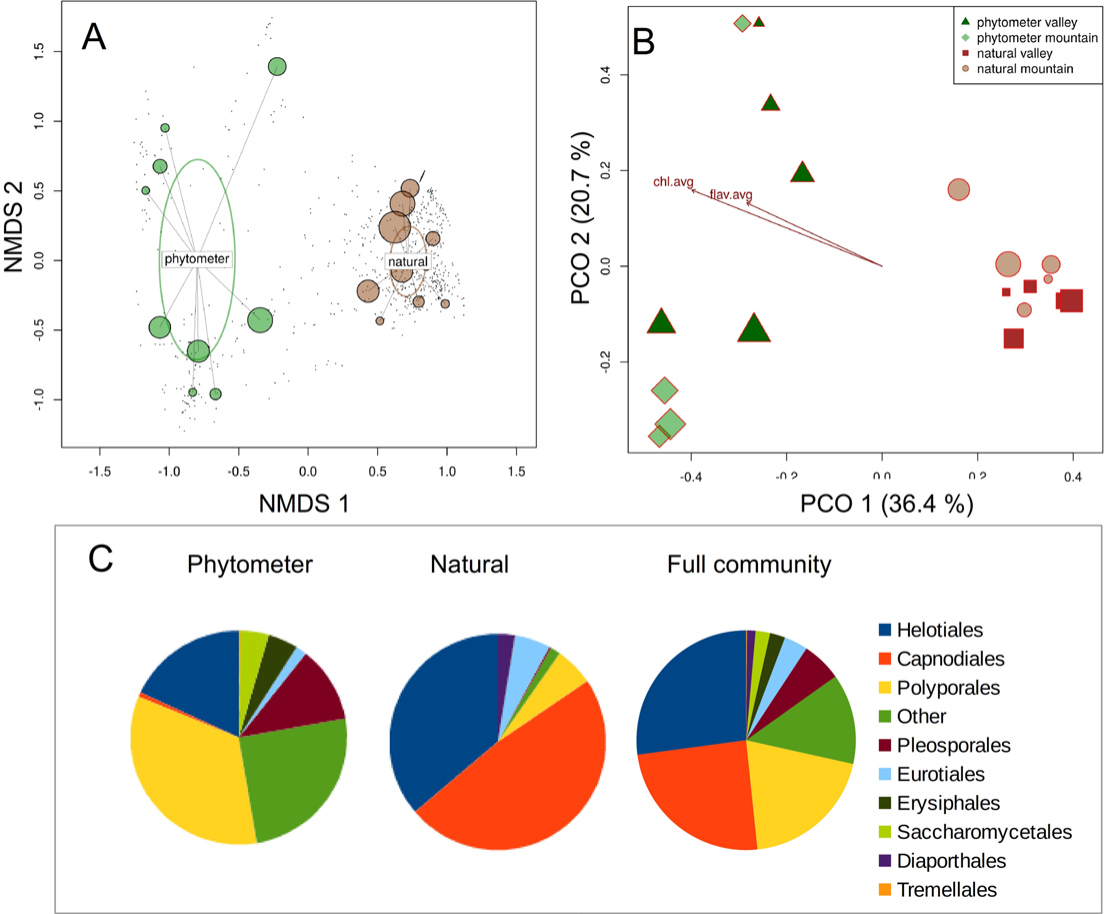
Differences in fungal community composition between phytometer and natural trees. [A] displays results from non-metric multidimensional scaling (NMDS) showing clearly separated phytometer and natural samples and a strong dispersion of the phytometer samples. [B] Principal coordinate analysis (PCO) confirmed this bisection into phytometer and natural fungal assemblages. It additionally displays those environmental parameters, which had a significant influence on fungal composition (chl.avg = chlorophyll content, flav.avg = flavonoid content). The higher dispersion of phytometer samples persisted in PCO and points towards a larger heterogeneity of the corresponding leaf mycobiome. [C] displays taxonomic composition of leaf-inhabiting fungi from phytometer trees, natural samples and the entire beech mycobiome for the nine most abundant orders (see text for the type of abundance measuring). All OTUs without taxonomic representation at the order level in the reference data set ("unidentified fungi") as well as less abundant orders (e.g. Botryosphaeriales, Diversisporales or Malasseziales) are combined into "Others".

Results from community analyses did not differ between complete and rarefied data (comparative analyses not shown, commands accessible via R-script). Community composition of leaf-inhabiting beech endophytes differed strongly between the phytometer and the naturally grown trees (Fig 4, Table 2). In both NMDS (Fig 4A, stress = 0.15, non-metric fit R^2^ = 0.977) and PCO (Fig 4B, 57.1% of total variance explained by the first two axes), the phytometer samples show a stronger dispersion than the natural samples. After analysis of valley and mountain data separately (Fig 4B), a significant difference in community composition was observed for the natural samples, but not for the phytometer trees (Table 2). The analysis of fungal taxonomic composition at order level added further insights into the differing fungal communities. In phytometer samples, the three most abundant orders in terms of maximal relative read abundance in any samples were the Polyporales (Basidiomycota; 33.9%), Helotiales (18.0%) and the Pleosporales (11.4%, both Ascomycota) with a considerable proportion of unidentified fungal OTUs (at the order level) and less abundant orders ("Other") (25.1%, Fig 4C, left). In samples from natural trees (Fig 4C, middle pie chart), the most dominant orders were the Capnodiales (48.3%), the Helotiales (36.1%) and the Eurotiales (5.7%) (all Ascomycota). The amount of unidentified and less abundant orders was markedly lower (< 5%).

**Table 2:** Statistical testing of fungal community composition in response to different environmental variables. The tests are based on a multispecies generalized linear model calculation (“Multispec.glm”) and a permutational multivariate analysis of variance using Bray-Curtis distance metrics (“Permanova”). All GLM tests resulted in the same highly significant p-values but the analysis of corresponding deviances indicated different responses to the environmental parameters. PERMANOVA yielded more sensitive results in all three displayed statistics and principally confirmed results from ordinations (Fig 4).

	Multispec. glm		Permanova		
	Dev	p	F	R^2^	p
Entire community					
Elevation	2864	**0.003**	7.03	0.23	**0.001**
Substrate	817	**0.003**	1.74	0.07	0.059
Elevation+Substrate					
Phytometer community					
Elevation	5.5	**0.003**	1.24	0.15	0.225
Chlorophyll	46.1	**0.003**	1.04	0.13	0.396
Flavonoids	46.1	**0.003**	1.29	0.16	0.239
Natural community					
Elevation	19.5	**0.003**	2.98	0.27	**0.007**
Chlorophyll	112	**0.003**	2.63	0.25	**0.004**
Flavonoids	102	**0.003**	2.71	0.25	**0.009**

### Correlation of leaf biochemistry and endophyte community

**Fig 5:**
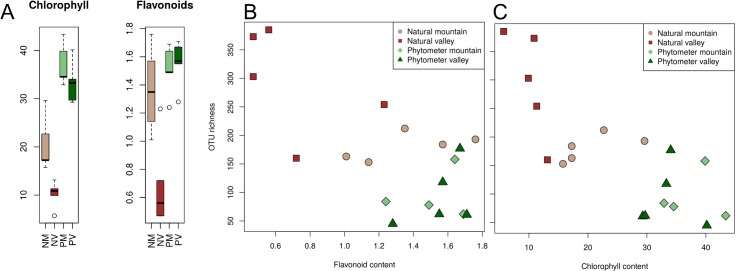
Chlorophyll and flavonoid contents of living leaves. [A] displays pigment contents (in μg cm^-2^) as box-whisker diagrams separated into the four different sample groups "natural mountain" (NM), "natural valley" (NV), "phytometer mountain" (PM) and "phytometer valley" (PV). [B and C] display relations of flavonoids and chlorophylls to OTU richness. Values differed significantly between phytometer and natural trees (KS test, D = 0.9, p < 0.01). The pigment concentrations were significantly higher in natural mountain than in natural valley samples (D = 1, p = 0.01), but did not differ within the phytometer samples (D = 0.55, p = 0.43).

We identified a strong correlation of leaf biochemistry with both site parameters and fungal biodiversity (Table 2). Statistical confidence increased when analysing the rarefied instead of the full community data (results not shown, analysis can be recapitulated from supplementary R-script). First, chlorophyll and flavonoid contents of the leaves were significantly higher in natural mountain samples than in natural valley samples (Fig 5). They were highest in leaves of the phytometer trees and lowest in samples from the natural valley trees. Second, OTU richness was inversely correlated with leaf biochemistry, such that leaves with highest chlorophyll and flavonoid concentrations revealed the lowest OTU richness (Fig 5B and 5C). Third, community composition was significantly correlated with leaf biochemistry (Fig 5B).

## Discussion

### The phytometer system

Experiments under modified (i.e. simplified) natural conditions have been conducted for decades and have resulted in the discovery of fundamental ecological principles [54–56]. In line with this research tradition, we established a field experiment near Berchtesgaden in the German Alps at two different altitudes and planted 100 two-years old young beech trees from a distant origin of each site. At those phytometer sites we investigated fungal biodiversity signals and dynamics under reduced environmental complexity (e.g. the trees are of identical age and were grown under identical conditions) since October 2013.

The present study is based on an initial sampling event immediately after planting of the phytometer trees. The newly introduced phytometer trees reflected their previous environment, a tree nursery in northern Germany, and additionally served as an independent control group to study the role of local biotic and abiotic parameters at different elevation for leaf mycobiomes of *F*. *sylvatica*.

### Assessment of the beech leaf mycobiome with NGS

Biological and ecological interpretation of fungal NGS data from environmental samples is generally complicated by the inability to distinguish between signals from extracellular DNA, DNA in dead hyphae or DNA from inactive spores. We used surface sterilised leaves for DNA extraction in order to create a strong focus on the endophytic part of the phyllosphere mycobiome, which is supposed to contain a higher proportion of living and active hyphae/cells compared with the mycobiomes from substrates with untreated surfaces [57].

The Illumina MiSeq platform was used to sequence full-length ITS1-5.8S-ITS2 rDNA amplicons [41]. Forward (R1) reads covered the entire ITS1 region, whereas reverse sequencing resulted in full-length ITS2 reads (R2). With the 300 base pairs paired-end sequencing, the overlap of R1 and R2 reads was too short and of too low quality to allow assembly of full-length ITS sequences. We decided to use the ITS1 data for biodiversity analysis, because ITS2 data were of generally lower quality and were therefore discarded in much higher quantity during demultiplexing. A thorough comparative analysis of ITS1 and ITS2 data is beyond the scope of this publication, however, a preliminary comparative analysis of ITS1 and ITS2 yielded congruent results of community signals, but seemed to differ slightly in taxonomic annotations (data not shown, but compare [58]).

### Phytometer and naturally grown trees differed in mycobiome diversity and composition as well as in leaf biochemistry

Generally speaking, the analyses of leaf mycobiomes as well as of leaf biochemistry clearly separated the phytometer from the natural trees. Our earlier raised hypothesis 1, that is a clear response of phyllosphere mycobiomes to local stand conditions of their host trees, cannot be rejected on the basis of the present data. This basic message did not change with rarefied data.

Although the daily handling of trees at the commercial tree nursery in northern Germany, from which the phytometer trees were obtained, falls under their corporate secret, we know from other comparable companies, that such trees are usually kept under most favourable growth conditions in optimized custom-made soils and with regular application of nutrients, fitness enhancers and protective agents. Additionally, aerial fungal spore concentration, the main tentative source of fungal leaf infections [3] might be strongly reduced in such tree nurseries due to permanent hygienic measures. Under such artificial conditions, fungal infections might be lower than in natural environments leading to lower infection rates and lower diversity (Fig 2C).

We additionally observed more heterogeneous and variable fungal assemblages of the phytometer samples (Fig 4A and 4B) and a more balanced taxonomic composition at order level (Fig 4C) than within leaves from the natural sites. In contrast to the recently observed community differences between natural trees from the valley and the mountain site [41], the phytometer valley and mountain samples were indistinguishable from each other. The corresponding GLM statistics (Table 2) missed sensibility in this case. The significance of their p-values would be certainly misleading, if used as sole measures of community turnover. One possible cause of differing fungal biodiversity signals between phytometer and natural trees certainly is the biochemical properties of the leaves [59].

The two leaf pigments chlorophyll and flavonoid had highest values in the phytometer trees suggesting an active photosynthetic machinery and thus durable plant tissues and a fully intact plant immune system [60]. Moreover, a significant correlation of fungal OTU richness (Fig 5B and 5C) and of community composition (Fig 4B) with those two leaf parameters has been observed. If we assume a direct connection of leaf pigment contents and biochemistry processes within the leaves, our results could point towards a close interconnectivity of leaf-inhabiting fungi with the tree host physiology [61, 62]. Possible causes of differing leaf pigments and mycobiome composition between phytometer and natural samples could be either a generally impaired or a stimulated plant defence under stressful conditions [63]. Undoubtedly, the naturally growing trees face the full climatic and ecological impacts of their unprotected natural environment and the trees' immunological barriers might be specifically adapted to the local environmental challenges. In such conditions the host trees might select for a more specialized, i.e. habitat-specific, phyllosphere fungal community. Our mycobiome "snapshot" seems to support this hypothesis, because fungal composition of natural trees was clearly skewed towards the two highly abundant orders Capnodiales and Helotiales whereas mycobiome composition of phytometer trees was clearly more balanced at ordinal level (Fig 4C).

The analysis of leaf pigments further revealed a significant increase from low to high elevation in natural samples (Fig 5A). These results were comparable to earlier pigment measurements of beech leaves, where higher chlorophyll contents were attributed to higher light intensity and availability [63]. Previous studies identified elevation (i.e. ambient temperature) as a predominant parameter shaping endophytic communities [21, 41, 64, 65]. Based on the present data we can only speculate about possible mechanisms of the observed correlations of elevation and mycobiome patterns. Further site conditions might determine leaf mycobiome composition. The different herbaceous vegetation for instance indicates different moisture content of the valley and mountain soils. Data gathering and analysis is ongoing and will soon allow for a more thorough validation of the present initial observations.

## Supporting Information

S1 Notes Illumina library. Step-by-step visualisation and description of sample multiplexing and structure of the Illumina amplicon library.
(PDF)

S2 Notes Biodiversity workflow in R. Bundle of files for biodiversity analysis in R. All necessary input files and a commented script of R-commands are provided.
(ZIP)

S1 Table Master data sheet. Spreadsheet file containing information about read abundances of operational taxonomic units (OTUs) and sample metadata. Here, data were prepared for subsequent biodiversity analysis in R.
(XLSX)

S1 Text Bioinformatics pipeline. Commented list of all commands used for demultiplexing and further sequence processing.
(PDF)
