# Supplementary material for: Diversity and Composition of the Leaf Mycobiome of Beech (Fagus sylvatica) Are Affected by Local Habitat Conditions and Leaf Biochemistry
Source: PLoS One. 2016 Apr 14;11(4):e0152878. doi: 10.1371/journal.pone.0152878 (PMC4831807; doi:10.1371/journal.pone.0152878)
Supplement: S1 Notes — Step-by-step visualisation and description of sample multiplexing and structure of the Illumina amplicon library. (PDF) [file pone.0152878.s001.pdf]

Illumina library preparation step-by-step: overview of pipetting scheme

Barcode combinations for Illumina library

		501-701	502-701	503-701	504-701	505-701	501-702	502-702	503-702	504-702	505-702	501-703	502-703
		1	2	3	4	5	6	7	8	9	10	11	12
**F1 – R1**	A	sample01	sample09	sample17	sample25	sample33	sample41	sample49	sample57	sample65	sample73	sample81	sample89
**F1 – R2**	B	sample02	sample10	sample18	sample26	sample34	sample42	sample50	sample58	sample66	sample74	sample82	sample90
**F1 – R3**	C	sample03	sample11	sample19	sample27	sample35	sample43	sample51	sample59	sample67	sample75	sample83	sample91
**F1 – R4**	D	sample04	sample12	sample20	sample28	sample36	sample44	sample52	sample60	sample68	sample76	sample84	sample92
**F2 – R1**	E	sample05	sample13	sample21	sample29	sample37	sample45	sample53	sample61	sample69	sample77	sample85	sample93
**F2 – R2**	F	sample06	sample14	sample22	sample30	sample38	sample46	sample54	sample62	sample70	sample78	sample86	sample94
**F2 – R3**	G	sample07	sample15	sample23	sample31	sample39	sample47	sample55	sample63	sample71	sample79	sample87	sample95
**F2 – R4**	H	sample08	sample16	sample24	sample32	sample40	sample48	sample56	sample64	sample72	sample80	sample88	sample96

This image shows possible combinations of tags (F1-R1, etc.) and indices (501-701, etc.) for Illumina sequencing of 96 samples.

The amplicon library is prepared with two consecutive amplification steps, which are described in the following two slides.

Illumina library preparation step-by-step: first amplification

- Illumina PCR 1 primer

Full-length Illumina sequencing primers

Name	Sequence
ITS1F_1	TACACGACGCTCTTCCGATCTTCATCTTGGTCATTTAGAGGAAGTAA
ITS1F_2	TACACGACGCTCTTCCGATCTAAGTGACTTGGTCATTTAGAGGAAGTAA
ITS1F_3	TACACGACGCTCTTCCGATCTTGCGAGACTTGGTCATTTAGAGGAAGTAA
ITS1F_4	TACACGACGCTCTTCCGATCTGACATCCACTTGGTCATTTAGAGGAAGTAA
ITS4_1	CAGACGTGTGCTCTTCCGATCAGGAGTCCTCCGCTTATTGATATGC
ITS4_2	CAGACGTGTGCTCTTCCGATCCGCTCATCCTCCGCTTATTGATATGC
ITS4_3	CAGACGTGTGCTCTTCCGATCGCTAACATCCTCCGCTTATTGATATGC
ITS4_4	CAGACGTGTGCTCTTCCGATCTTGACCAGTCCTCCGCTTATTGATATGC

- Amplicon structure after Illumina PCR 1

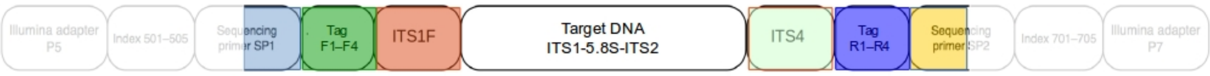

This first PCR is done as a conventional ITS amplification.

It adds a first pair of barcode sequences (“F” and “R” tags) to the samples and half of the Illumina-specific sequencing primer sequence as a binding site for PCR 2 primers (next slide)

The maximal number of possible tag combinations is 16 (F1-R1, F1-R2, F1-R3, F1-R4, F2-R1, etc.).

If more than 16 samples are prepared for sequencing, identical tag combinations will be used for more than one sample (see pipetting scheme of slide one.

The high multiplexing capacity comes from the second amplification step, which introduces another pair of barcodes (“500” and “700” indices, see next slide).

Illumina library preparation step-by-step: second amplification

- Template for Illumina PCR 2

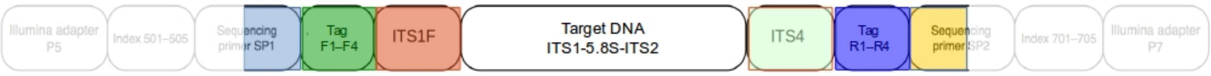

- Illumina PCR 2 primer

Full-length Illumina sequencing primers	
**Name**	**Sequence**
PSP_501	AATGATACGGCGACCACCGAGATCTACACTAGATCGCACACTCTTTCCCTACACGACGCTCTTCCGATCT
PSP_502	AATGATACGGCGACCACCGAGATCTACACCTCTCTATACACTCTTTCCCTACACGACGCTCTTCCGATCT
PSP_503	AATGATACGGCGACCACCGAGATCTACACTATCCTCTACACTCTTTCCCTACACGACGCTCTTCCGATCT
PSP_504	AATGATACGGCGACCACCGAGATCTACACAGAGTAGAACACTCTTTCCCTACACGACGCTCTTCCGATCT
PSP_505	AATGATACGGCGACCACCGAGATCTACACGTAAGGAGACACTCTTTCCCTACACGACGCTCTTCCGATCT
PSP_701	CAAGCAGAAGACGGCATACGAGATTAAGGCGAGTGACTGGAGTTCAGACGTGTGCTCTTCCGATC
PSP_702	CAAGCAGAAGACGGCATACGAGATCGTACTAGGTGACTGGAGTTCAGACGTGTGCTCTTCCGATC
PSP_703	CAAGCAGAAGACGGCATACGAGATAGGCAGAAGTGACTGGAGTTCAGACGTGTGCTCTTCCGATC
PSP_704	CAAGCAGAAGACGGCATACGAGATTCCTGAGCGTGACTGGAGTTCAGACGTGTGCTCTTCCGATC
PSP_705	CAAGCAGAAGACGGCATACGAGATGGACTCCTGTGACTGGAGTTCAGACGTGTGCTCTTCCGATC

- Amplicon structure after Illumina PCR 2 ready for sequencing

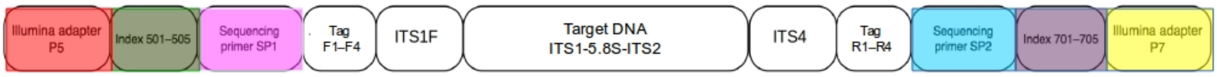

This second PCR is done only for adding the index pairs, the Illumina adaptors and for completing the Illumina-specific sequencing primer site.

This amplification runs for only 5 cycles, in order to keep primer dimer formation low. The maximal number of possible index combinations is 25 (501[2-5]-701[2-5]).

Thus, the maximum number of samples is 400 (16 * 25)

Illumina library preparation step-by-step: Processing of Illumina reads (demultiplexing)

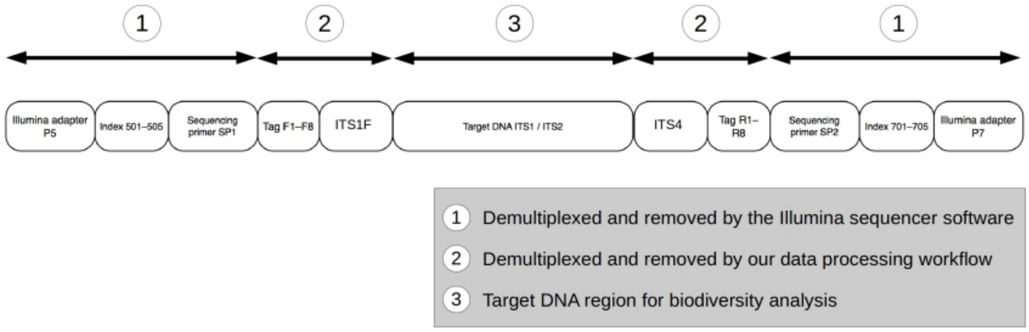

Demultiplexing is achieved in two steps.
The Illumina software recognises the index combinations and separates the corresponding reads into different fastq files.
It also removes the adaptors, indices and sequencing primers.
The reads therfore begin with either forward or reverse tags and enter our custom-made sequence processing workflow, that, among others, contains the second demultiplexing step.
During demultiplexing, all four indices/tags were recognized from the read pairs and separated into individual, sample-based fasta files.
