# Supplementary material for: Diversity and Composition of the Leaf Mycobiome of Beech (Fagus sylvatica) Are Affected by Local Habitat Conditions and Leaf Biochemistry
Source: PLoS One. 2016 Apr 14;11(4):e0152878. doi: 10.1371/journal.pone.0152878 (PMC4831807; doi:10.1371/journal.pone.0152878)
Supplement: S1 Text — Commented list of all commands used for demultiplexing and further sequence processing. (PDF) [file pone.0152878.s004.pdf]

The following document describes in detail the sequence processing and demultiplexing workflow which consists of tag identification, quality filtering, sample splitting, OTU picking, taxon assignment, OTU table making, alignment and phylogeny.

1) Extracting the tags (barcodes) from the read pairs

```
qiime > extract_barcodes.py -f 501-701_R1.fastq -r 501-
701_R2.fastq -c barcode_paired_end --bc1_len 8 --bc2_len 8 -o
Step01/501-701_extract_barcodes
```

This command is applied for all existing index combinations, like 501-702, 501-703, 501-704, etc.

2) Recognition of the tags and quality filtering

```
qiime > split_libraries_fastq.py -i Step01/501-
701_extract_barcodes/reads1.fastq -o Step02/501-
701_split_libraries/R1/ -m mapping_file.txt -b Step01/501-
701_extract_barcodes/barcodes.fastq -q 29 --barcode_type 16

qiime > split_libraries_fastq.py -i Step01/501-
701_extract_barcodes/reads2.fastq -o Step02/501-
701_split_libraries/R2/ -m mapping_file.txt -b Step01/501-
701_extract_barcodes/barcodes.fastq -q 29 --barcode_type 16
```

Again, this procedure continues for all existing index combinations

3) Extracting sample-specific reads into different fasta files

```
qiime > extract_seqs_by_sample_id.py -i Step02/501-
701_split_libraries/R1/seqs.fna -o Your_study/sample01_R1.fasta -m
mapping_file.txt -s "SampleID:f1r1"
qiime > extract_seqs_by_sample_id.py -i Step02/501-
701_split_libraries/R2/seqs.fna -o Your_study/sample01_R2.fasta -m
mapping_file.txt -s "SampleID:f1r1"
```

This procedure continues until all samples were demultiplexed and respective fasta sequences were generated. Naming of output files (-o option) and of -s option strongly depends on the pipetting scheme of the library and therefore of the contents of the mapping file. In the examples above, R1 and R2 read pair of sample 1 is defined by the index/tag combination 501-f1-r1-701.

It is recommended to separate the fasta files according to different research projects. Here, the Folder "Your_study" was named "Beech_Leaf_Mycobiome"

In general, R2 reads were obtained in lower base calls, the stringent quality filtering therefore discarded more reverse reads than forward reads. the following steps are shown for forward reads (ITS1 region) only, but can be applied to R2 reads accordingly.

4) Renaming of fasta sequence headers to ascending counts

This command requires the installation of the fastx toolkit (http://hannonlab.cshl.edu/fastx_toolkit/index.html, last accessed June 2015)

```
fastx_renamer -n COUNT -i Your_study/sample01_R1.fasta -o Step04/sample01_R1_short.fasta

fastx_renamer -n COUNT -i Your_study/sample02_R1.fasta -o Step04/sample02_R1_short.fasta
```

This procedure continues until all fasta files were processed

5) Adding file names (sampleID) to the sequence headers:

cd to path/of/your/fasta/files, then run

```
perl rename.pl
```

The file "rename.pl" is available as supporting material from Bálint, Miklós, Philipp-André Schmidt, Rahul Sharma, Marco Thines, and Imke Schmitt. “An Illumina Metabarcoding Pipeline for Fungi.” Ecology and Evolution 4, no. 13 (July 2014): 2642–53. doi:10.1002/ece3.1107.
This procedure generates a new set of fasta files with the file name (e.g. sample01_R1_short.fasta) added to the sequence header.

6) Merging fasta files

```
cat renamed_*short.fasta >> your_study_all_R1.fasta
```

With the simple unix call 'cat', all sample-specific fasta files were merged.

7) Removing of conserved flanking regions with the FungalITSextractor

This freely available tool is available from http://www.emerencia.org/FungalITSextractor.html. For our ITS1 reads, flanking SSU and 5.8S regions were removed. The ITS extractor might be replaced by the newer and improved tool ITSx: http://microbiology.se/software/itsx/

8) OTU picking:

```
qiime > pick_otus.py -s 0.97 -i your_study_all_R1_trim.fasta -o Your_study_OTUs -m usearch -x
```

9) Picking of representative sequences:

```
qiime > pick_rep_set.py -i Your_study_OTUs/your_study_all_R1_trim_otus.txt -f your_study_all_R1_trim.fasta -o Your_study_OTUs/your_study_all_R1_trim_repset.fasta
```

10) Taxon assignment of the representative sequences

```
qiime > assign_taxonomy.py -i Your_study_OTUs/your_study_all_R1_trim_repset.fasta -o Your_study_Taxonomy -m blast -r /Path/to/sh_refs_qiime_ver6_dynamic_s_04.07.2014.fasta -t /Path/to/sh_taxonomy_qiime_ver6_dynamic_s_04.07.2014.txt
```

11) Making several OTU tables and taxon summaries

```
qiime > make_otu_table.py -i
Your_study_OTUs/your_study_all_R1_trim_otus.txt -t
Your_study_Taxonomy/your_study_all_R1_trim_repset_tax_assignments.
txt -o Your_study_OTUs/your_study_all_R1_trim.biom
```

```
qiime > summarize_taxa.py -i
Your_study_OTUs/your_study_all_R1_trim.biom -o
Your_study_Taxonomy_Summary
```

See how to analyse the summary files: http://www.wernerlab.org/teaching/qiime/overview/c

```
biom summarize-table -i
Your_study_OTUs/your_study_all_R1_trim.biom -o
Your_study_Taxonomy_Summary/your_study_all_R1_trim_summary_otu_tab
le.txt
```

```
qiime > make_otu_heatmap_html.py -i
Your_study_OTUs/your_study_all_R1_trim.biom -o
Your_study_OTUs/Heatmap/
```

```
biom convert -i Your_study_OTUs/your_study_all_R1_trim.biom -o
Your_study_OTUs/your_study_all_R1_trim_otu_final.txt -b --header-
key taxonomy --output-metadata-id "taxonomy"
```

12) Making reference alignment with MAFFT

```
mafft  --genafpair  --maxiterate 16 --inputorder
/path/to/your_study_all_R1_trim_repset.fasta >
/path/to/your_study_all_R1_trim_repset_mafft.fasta
```

Mafft was installed from http://mafft.cbrc.jp/alignment/software/

13) Final alignment and phylogeny

```
qiime > align_seqs.py -i
/path/to/your_study_all_R1_trim_repset.fasta -m mafft -a mafft
-t /path/to/your_study_all_R1_trim_repset_mafft.fasta -o
/path/to/your_study_all_R1_trim_repset_mafft.fasta
```

```
qiime > make_phylogeny.py -i
/path/to/your_study_all_R1_trim_repset_mafft.fasta
```
